# Hepatic Overexpression of Lysophospholipid Acyltransferase LPLAT14/MBOAT1 Ameliorates Mild Liver Injury During Early High‐Fat Diet Feeding

**DOI:** 10.1096/fba.2026-00165

**Published:** 2026-11-02

**Authors:** Sho Hori, Kahori Shimizu, Taiga Kikuchi, Hajime Miyata, Hiroaki Hase, Hiroki Nakanishi, Hideo Shindou, Hiroyuki Mizuguchi

**Affiliations:** ^1^ Laboratory of Biochemistry and Molecular Biology, Graduate School of Pharmaceutical Sciences The University of Osaka Osaka Japan; ^2^ Laboratory of Biochemistry and Molecular Biology, School of Pharmaceutical Sciences The University of Osaka Osaka Japan; ^3^ Lipidome Lab Co., Ltd. Akita Japan; ^4^ Department of Lipid Life Science, National Institute of Global Health and Medicine Japan Institute for Health Security Tokyo Japan; ^5^ Department of Medical Lipid Science, Graduate School of Medicine The University of Tokyo Tokyo Japan; ^6^ Global Center for Medical Engineering and Informatics The University of Osaka Osaka Japan; ^7^ Integrated Frontier Research for Medical Science Division, Institute for Open and Transdisciplinary Research Initiatives The University of Osaka Osaka Japan; ^8^ Center for Infectious Disease Education and Research The University of Osaka Osaka Japan

**Keywords:** adenovirus vector, liver injury, LPLAT14, lysophospholipid acyltransferase, oleic acid, phosphatidylserine, phospholipid

## Abstract

Metabolic dysfunction‐associated steatotic liver disease (MASLD) encompasses a spectrum of conditions progressing from simple steatosis to severe steatohepatitis. During this progression, liver injury associated with lipotoxicity and altered membrane phospholipid homeostasis is thought to contribute to disease aggravation. Although lysophospholipid acyltransferases (LPLATs) regulate membrane lipid remodeling and contribute to cellular integrity, the physiological roles of several LPLATs have only partially been characterized, and the in vivo functions of LPLAT14 remain largely unknown. Here, we investigated the effects of hepatic overexpression of LPLAT14 on high‐fat diet (HFD)‐induced metabolic disorders and mild liver injury in mice using an improved adenovirus (Ad) vector. Hepatic LPLAT14 overexpression did not significantly alter body weight, systemic glucose tolerance, or hepatic lipid accumulation. Targeted lipidomics revealed that LPLAT14 overexpression selectively significantly increased oleic acid (OA)‐containing phosphatidylserine (PS) species in the liver, suggesting selective incorporation of OA into lysophosphatidylserine. This specific lipidomic remodeling significantly attenuated mild liver injury during early HFD feeding, as evidenced by reduced serum alanine aminotransferase levels at 2 weeks after Ad‐LPLAT14 treatment. However, this hepatoprotective effect was not observed in the later phase (8 weeks), when severe lipid accumulation had developed, suggesting that LPLAT14 primarily attenuates mild liver injury during early HFD feeding. Collectively, our findings suggest that LPLAT14‐mediated enrichment of OA‐containing PS attenuates mild liver injury during early HFD feeding, possibly through protection against early cellular stress.

## Graphical abstract

Using an improved adenovirus vector, we investigated the effects of hepatic LPLAT14 overexpression on high‐fat diet (HFD)‐induced metabolic disorders and mild liver injury in mice. LPLAT14 selectively increased oleic acid (OA)‐containing phosphatidylserine (PS) species in the liver. This specific lipidomic remodeling attenuated mild liver injury during early HFD feeding, evidenced by reduced serum alanine aminotransferase levels. Our findings suggest that LPLAT14‐mediated enrichment of OA‐containing PS attenuates early HFD‐induced liver injury, possibly through protection against early cellular stress.

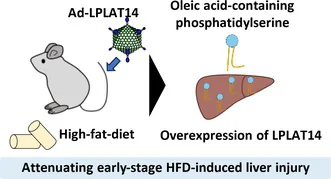

## 1 Introduction

The global prevalence of lifestyle‐related diseases, such as obesity, Type 2 diabetes, and metabolic dysfunction‐associated steatotic liver disease (MASLD), has increased markedly in recent years [1, 2]. Notably, MASLD is not merely a benign condition but encompasses a spectrum of liver diseases; without appropriate intervention, it can progress from simple steatosis to metabolic dysfunction‐associated steatohepatitis (MASH), fibrosis, and ultimately hepatocellular carcinoma [3, 4]. Excessive dietary lipid intake is a primary environmental factor contributing to these metabolic disorders [5]. Chronic consumption of a HFD disrupts systemic lipid homeostasis, leading to various deleterious effects, including lipotoxicity, chronic inflammation, and insulin resistance in metabolic organs, such as the liver and adipose tissue [6, 7, 8]. In this context, the pathogenesis of liver injury is recognized as a complex process that extends far beyond the simple accumulation of ectopic fat. Emerging evidence has suggested that qualitative alterations in the hepatic lipidome contribute to hepatocyte damage [9, 10]. Specifically, the intracellular accumulation of lipotoxic intermediates, coupled with alterations in membrane phospholipid homeostasis, impairs cellular function and viability [11, 12]. As HFD‐induced liver injury is orchestrated by this intricate and multifactorial interplay of lipotoxicity and structural membrane remodeling, establishing effective pharmacological interventions remains a formidable challenge [4, 13].

Lipid homeostasis is partly maintained by phospholipids, which are the major components of cellular membranes and consist of a glycerol backbone, two fatty acyl chains, and a polar head group. Phospholipids are distinguished by the specific combination of their polar head groups and fatty acyl chains. The vast diversity of fatty acid compositions in phospholipids—differing in carbon chain length and the number and position of double bonds—is primarily generated through a remodeling pathway known as the Lands cycle [14, 15]. In this cycle, phospholipids are cleaved by phospholipase A1/2 to form lysophospholipids, which are subsequently remodeled back into phospholipids by lysophospholipid acyltransferases (LPLATs) [16, 17, 18]. This specific fatty acid composition determines membrane properties and biological functions; consequently, its dysregulation is closely linked to various diseases, including diabetes mellitus and MASLD [19, 20]. To date, 14 mammalian LPLATs have been identified, and their physiological importance has been established. For instance, our previous study demonstrated that liver‐specific LPLAT10 overexpression increases DHA‐containing PC, thereby enhancing postprandial insulin secretion and suppressing postprandial hyperglycemia [21]. In addition, Hishikawa et al. showed that liver‐specific deletion of LPLAT3, also known as 1‐acyl‐sn‐glycerol‐3‐phosphate acyltransferase 3 (AGPAT3), reduces hepatic DHA‐containing phospholipids, which in turn activates SREBP1 processing to drive the compensatory synthesis and systemic delivery of polyunsaturated fatty acids [22]. Thus, LPLATs play crucial roles in various biological processes and pathological conditions.

Among the LPLAT family members, LPLAT14, also known as membrane‐bound glycerophospholipid O‐acyltransferase 1 (MBOAT1) or lysophosphatidylcholine acyltransferase 1 (LPEAT1), reports regarding its pathophysiological roles remain scarce. In vitro studies have characterized LPLAT14 as an enzyme that preferentially incorporates oleic acid (OA) into lysophosphatidylethanolamine (LPE) and stearic acid and OA into lysophosphatidylserine (LPS) [23, 24]. Recent findings have suggested that LPLAT14 plays a crucial role in inhibiting ferroptosis, an iron‐dependent form of regulated cell death, by modulating membrane lipid composition [25, 26, 27]. For example, high LPLAT14 expression in cancer cells reduces membrane polyunsaturated fatty acid levels and inhibits ferroptosis in a GPX4‐independent manner [28, 29, 30]. Although the specific combinations of lysophospholipids and fatty acids utilized by LPLAT14 have been investigated in vitro, their specific combinations and physiological functions in vivo remain largely unclear. In addition, previous reports have indicated that LPLAT14 is scarcely expressed in the liver under physiological conditions [23]. Consequently, the role of LPLAT14 in the regulation of hepatic metabolism, liver injury, and phospholipid remodeling, particularly under HFD‐induced pathological conditions, remains unclear.

In this study, we investigated the effects of hepatic LPLAT14 overexpression on HFD‐induced glucose and lipid metabolic disorders and liver injury in mice. To overexpress LPLAT14 in the liver, we selected an adenovirus (Ad) vector because the systemic administration of Ad vectors results in liver‐specific expression of exogenous genes [31]. Among the Ad vectors, we utilized an improved Ad vector named Ad‐E4‐122aT [32, 33], which exhibits higher and longer‐term transgene expression and lower hepatotoxicity than conventional Ad vectors.

## 2 Materials and Methods

### 2.1 Mice and Cells

Six‐week‐old male C57BL/6N mice were obtained from Nippon SLC (Hamamatsu, Japan). An HFD (32, 56.7% kcal from fat; the fatty acid composition is Supplemental Figure 1) was obtained from CLEA Japan (Tokyo, Japan). Ad vectors were intravenously administered to mice at 5 × 10^9^ infectious units (IFU) per mouse via the tail vein, and the mice were simultaneously fed an HFD. Mice were anesthetized by inhalation of isoflurane (3%) (FUJIFILM Wako Pure Chemical Corporation, Osaka, Japan) and then euthanized for tissue collection. No relevant adverse effects were observed in the experimental animals. The mice were maintained on a 12/12 h light/dark cycle and provided free access to water and food. Body weight was measured weekly. All efforts were made to minimize animal suffering. HEK293 cells were cultured in Dulbecco's modified Eagle's medium (FUJIFILM Wako Pure Chemical Corporation, Osaka, Japan) supplemented with 10% FBS, 2 mmol/L L‐L‐alanyl‐L‐glutamine (Nacalai Tesque, Kyoto, Japan), and antibiotics.

### 2.2 Plasmids and Ad Vectors

Ad vectors were constructed using an improved in vitro ligation method [34, 35]. LPLAT14 (NM_153546.5) complementary DNA (cDNA) was cloned by PCR amplification from stomach‐derived cDNA obtained from C57BL/6 mouse. The PCR fragment was first cloned into pHMCA6 and sequenced [36]. The AHA promoter (liver‐specific apolipoprotein E enhancer‐hepatocyte control region‐human α1‐antitrypsin promoter)‐driven LPLAT14‐expression plasmid, pAHA‐LPLAT14, was constructed using pAHA [32]. pAHA‐LPLAT14 was digested with I‐*Ceu*I/PI‐*Sce*I. Fragments of pAHA‐LPLAT14 were ligated into the I‐CeuI/PI‐SceI‐digested Ad vector plasmid, pAdHM4‐E4‐122aT [32, 33], to generate pAd‐AHA‐LPLAT14. The firefly luciferase‐expressing plasmid pAd‐AHA‐Luc, driven by the AHA promoter, was used as a control. pAd‐AHA‐LPLAT14 and pAd‐AHA‐Luc were digested with *Pac*I and transected into HEK293 cells using Lipofectamine 3000 (Thermo Fisher Scientific, Carlsbad, CA, USA), producing Ad‐LPLAT14 and Ad‐Luc, respectively. All ad vectors (Ad‐LPLAT14 and Ad‐Luc) were purified as previously described [32]. Virus particles were determined using a spectrophotometric method [37], and biological titers were measured using an Adeno‐X‐rapid titer kit (Clontech, Mountain View, CA, USA). The ratio of the particle‐to‐biological titer was approximately 6 for the purified Ad vectors used in this study.

### 2.3 Quantitative Reverse Transcription Polymerase Chain Reaction (RT‐PCR) Analysis of Gene Expression in Mouse Tissues

Total RNA was extracted from mouse tissues using ISOGEN (Nippon Gene, Tokyo, Japan) according to the manufacturer's instructions. Total pancreatic RNA was isolated as described previously [38]. mRNA levels were determined using quantitative RT‐PCR with THUNDERBIRD NEXT SYBR qPCR Mix (TOYOBO, Osaka, Japan) and normalized to β‐actin mRNA levels. The following thermal cycling protocol was used: 60 s at 95°C, followed by 40 cycles of 15 s at 95°C and 60 s at 63°C. The sequences of the primers used in this study were as follows: β‐actin, forward 5′‐GGCTGTATTCCCCTCCATCG‐3′ and reverse 5′‐CCAGTTGGTAACAATGCCATGT‐3′; and LPLAT14, forward 5′‐CAGTCGAGCCAACCATCAGT‐3′ and reverse 5′‐CTCCGAGACTGCCTTTGAGG‐3′.

### 2.4 Biochemical Analysis

Blood samples were collected from fasted (16 h) mice through retro‐orbital bleeding, and serum was obtained by centrifugation. Serum triglyceride (TG), total cholesterol (CHO), alanine aminotransferase (ALT), and lactate dehydrogenase (LDH) levels in mice were determined using Fuji DRI‐CHEM 4000v (Fujifilm, Tokyo, Japan). Serum free fatty acid (FFA) levels in mice were determined using LabAssay (TM) NEFA (FFA) (FUJIFILM Wako Pure Chemical Corporation, Osaka, Japan). Serum insulin levels in mice were determined using an Ultrasensitive Mouse Insulin ELISA kit (Mercodia, Uppsala, Sweden). Insulin resistance was evaluated using the homeostatic model assessment for insulin resistance (HOMA‐IR). HOMA‐IR was calculated according to the following formula: HOMA‐IR = fasting glucose (mg/dL) × fasting insulin (μU/mL)/405. In addition, fasting blood glucose levels were measured in tail blood collected from fasted (16 h) mice using a Glutest Sensor Neo (Sanwa Kagaku Kenkyusho, Nagoya, Japan).

### 2.5 Glucose Tolerance Tests

Glucose tolerance tests were performed on 16‐h‐fasted mice following intraperitoneal injection of 1 g/kg glucose. Blood glucose levels were determined immediately before and at the indicated times after injection using the Glutest Sensor Neo.

### 2.6 Hematoxylin and Eosin (H&E) Staining

Liver was fixed in 4% paraformaldehyde (FUJIFILM Wako Pure Chemical Corporation). The samples were embedded in paraffin at Applied Medical Research (Osaka, Japan). Paraffin sections (5 μm) were deparaffinized in xylene (FUJIFILM Wako Pure Chemical Corporation), rehydrated, and stained with the standard H&E protocol. Images were captured using a SLIDEVIEW VS200 Universal Whole Slide Imaging Scanner (EVIENT, Tokyo, Japan).

### 2.7 Oil Red O Staining

Liver tissues were fixed in 4% paraformaldehyde (FUJIFILM Wako Pure Chemical Corporation) for 24 h, embedded in OCT compound (Sakura Finetek, Tokyo, Japan), and snap‐frozen. Cryosections (10 μm) were fixed with 4% paraformaldehyde for 15 min, rinsed with water and 60% isopropanol (Nacalai Tesque, Kyoto, Japan), and stained with Oil Red O working solution for 15 min. Sections were counterstained with hematoxylin to visualize nuclei. Images were captured using a SLIDEVIEW VS200 Universal Whole Slide Imaging Scanner. The Oil Red O‐stained areas were quantified using ImageJ software (NIH, Bethesda, MD, USA). The percentage of Oil Red O‐positive area relative to the total tissue area was calculated from at least six randomly selected fields per single tissue section for each mouse.

### 2.8 Phospholipid Analysis

For the targeted analysis of phosphatidylethanolamine (PE), lysophosphatidylethanolamine (LPE), PS, and lysophosphatidylserine (LPS), lipidome analysis was conducted according to the Lipidome Lab Non‐targeted Lipidome Scan package (Lipidome Lab, Akita, Japan), using liquid chromatograph‐Orbitrap mass spectrometry (LC‐Orbitrap MS) based on the methods described previously [39, 40]. Briefly, the sample was dissolved with methanol and then homogenized. Total lipids were extracted using the liquid–liquid extraction as the Bligh & Dyer method. An aliquot of the lower/organic phase was evaporated to dryness under nitrogen gas, and the residue was re‐dissolved in methanol for LC–MS/MS measurements. Liquid chromatography (LC)‐electrospray ionization‐MS/MS analysis was performed by using a Q‐Exactive Plus mass spectrometer with an UltiMate 3000 LC system (Thermo Fisher Scientific). Samples were separated on an L‐column3 C18 metal‐free column (2.0 μm, 2.0 mm × 100 mm i.d.) at 40°C using a gradient solvent system: mobile phase A (isopropanol/methanol/water (5/1/4 v/v/v) supplemented with 5 mM ammonium formate and 0.05% ammonium hydroxide (28% in water))/mobile phase B (isopropanol supplemented with 5 mM ammonium formate and 0.05% ammonium hydroxide [28% in water]) ratios of 60%/40% (0 min), 40%/60% (0–1 min), 20%/80% (1–9 min), 5%/95% (9–11 min), 5%/95% (11–22 min), 95%/5% (22–22.1 min), 95%/5% (22.1–25 min), 60%/40% (25–25.1 min), and 60%/40% (25.1–30 min). The injection volume was 10 μL, and the flow rate was 0.1 mL/min. A heated electrospray ionization (HESI‐II) source conditions were as follows: ionization mode, positive or negative; sheath gas, 60 arbitrary units; auxiliary gas, 10 arbitrary units; sweep gas, 0 arbitrary units; spray voltage, 3.2 kV in positive and −3.0 kV in negative mode; heater temperature, 325°C; ion transfer capillary temperature, 300°C in positive and −320°C in negative mode; and S‐lens RF level, 50. The Orbitrap mass analyzer was operated at a resolving power of 70,000 in full‐scan mode (scan range 200–1800 m/z in positive and negative mode; automatic gain control (AGC) target 1e6 in positive and 3e6 in negative mode) and a resolving power of 17,500 in positive and 35,000 in negative mode in the Top 20 data‐dependent MS2 mode (stepped normalized collision energy 20, 30, and 40; isolation window 4.0 m/z; AGC target 1e5) with a dynamic exclusion setting of 10.0 s.

Post‐processing of the raw data files was done using the lipid molecular identification software, Lipid Search 5.1.6 (Mitsui Knowledge Industries Co. Ltd., Japan), which identifies individual intact lipid molecules on the basis of their molecular weight and fragmentation patterns from headgroup and fatty acid composition. In this method, biological matrix effects cannot be normalized in all detected peaks, because it is not possible to prepare appropriate internal standards corresponding to all the detected peaks. The relative values were calculated using the ratio of the chromatographic peak area of each analyte to that of the total analyte. On the other hand, for some lipid classes, values were calculated by normalizing them using internal standards. In addition, the quantification and annotation method used in this study corresponds to “absolute quantification Level 2 or 4” and “Fatty Acyl/Alkyl Level or Hydroxyl Group Level” defined by the Lipidomics Standard initiative, respectively [41]. All internal standard reagents were purchased from Avanti Polar Lipids (Alabaster, AL, USA). Methanol, isopropanol, and chloroform of ultra‐performance liquid chromatography (UPLC)/MS quality were obtained from FUJIFILM Wako Pure Chemical Corporation (Osaka, Japan). Ultrapure water was obtained from a Milli‐Q water system (Millipore, Milford, MA, USA).

The analysis of phosphatidylcholine (PC) and lysophosphatidylcholine (LPC) was performed using a distinct protocol. Briefly, pre‐treated liver samples were analyzed using a Nexera X3 Ultra‐high‐performance liquid chromatography (UHPLC) system coupled with an LC‐MS‐8060NX triple quadrupole mass spectrometer (Shimadzu Corporation, Kyoto, Japan). The UHPLC mobile phases, gradient conditions, and detection of individual phospholipid species were conducted using a semi‐targeted LC‐MS/MS approach based on the MRM Library for Phospholipid Profiling (Shimadzu Corporation). Peak alignment and peak height quantification were performed using Traverse MS software (Shimadzu Corporation).

### 2.9 Statistical Analysis

Statistical analyses were performed using GraphPad Prism (GraphPad Software, San Diego, California, USA). The Mann–Whitney *U* test was used to compare the differences between two independent groups, and one‐way analysis of variance (ANOVA) with Dunnett's post hoc test was used for multiple comparisons. Data are presented as mean ± standard error (SE), and a *p* < 0.05 was considered statistically significant.

## 3 Results

### 3.1 Tissue Distribution of LPLAT14 and Its Liver‐Specific Overexpression Using an Improved Ad Vector

To examine the tissue distribution of LPLAT14, LPLAT14 mRNA levels were determined in various tissues of male C57BL/6 mice. LPLAT14 mRNA was primarily expressed in the stomach, colon, epididymal adipose tissue, and skeletal muscle, whereas its expression was relatively low in most other organs, including the liver (Figure 1A).

**FIGURE 1:**
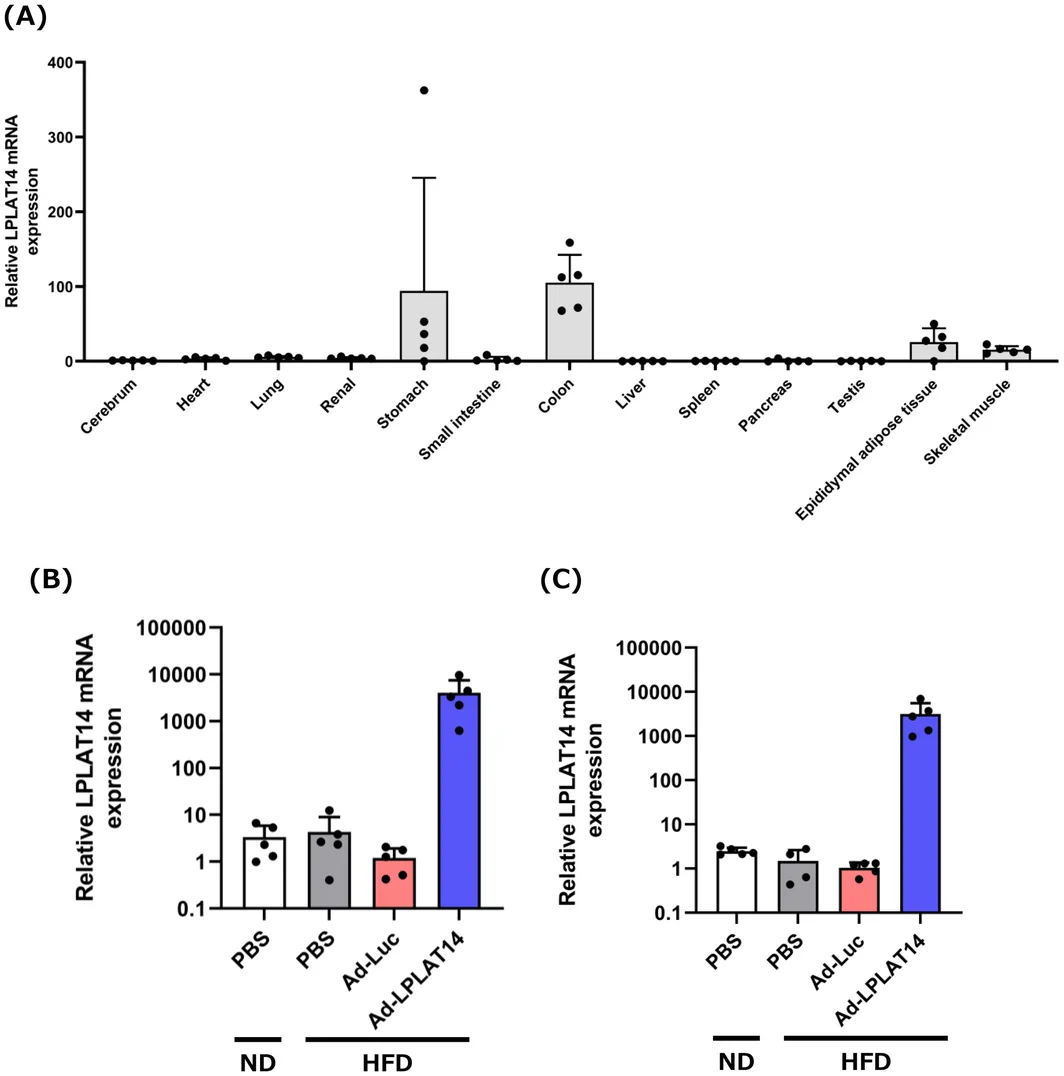
Tissue distribution of lysophospholipid acyltransferase (LPLAT)14 mRNA expression and hepatic overexpression of LPLAT14 using an adenoviral (Ad) vector. (A) LPLAT14 mRNA levels in tissues from C57BL/6 mice, measured using quantitative reverse transcription polymerase chain reaction (RT‐PCR). LPLAT14 mRNA expression in the colon was set to 100. Hepatic LPLAT14 mRNA levels at (B) 2 and (C) 8 weeks after administration of an LPLAT14‐expressing Ad vector (Ad‐LPLAT14), a luciferase‐expressing Ad vector (Ad‐Luc), or phosphate‐buffered saline (PBS), as determined by quantitative RT‐PCR. Hepatic LPLAT14 mRNA expression in Ad‐Luc‐treated mice was set to 1.0. Two independent experiments were performed and yielded similar results. Data are expressed as the mean ± SE (*n* = 4–5).

To overexpress LPLAT14 in the mouse liver, an LPLAT14‐expressing Ad vector (Ad‐LPLAT14) or a luciferase‐expressing Ad vector (Ad‐Luc), a control Ad vector, was administered intravenously to C57BL/6 mice, followed by feeding with an HFD. At 2 and 8 weeks after administration of the Ad vector, hepatic expression of LPLAT14 in Ad‐LPLAT14‐treated mice was approximately 2900‐ and 2500‐fold higher, respectively, than that in Ad‐Luc‐treated mice (Figure 1B,C).

### 3.2 Hepatic LPLAT14 Overexpression Selectively Increases OA‐Containing PS

Next, we investigated the effect of hepatic LPLAT14 overexpression on hepatic phospholipid composition. Based on previous in vitro studies showing that LPLAT14 exhibits acyltransferase activity toward LPE and LPS, LC‐MS/MS analysis was performed to profile PE, LPE, PS, and LPS species. In addition, the fatty acid composition of PE and PS was analyzed to determine whether LPLAT14 preferentially affects the incorporation of specific fatty acids into these phospholipids in vivo. No significant differences were observed in the overall composition of PE and LPE species between the livers of Ad‐LPLAT14‐ and Ad‐Luc‐treated mice (Figure 2A,B). Consistently, no clear fatty acid selectivity was observed for PE (Figure 2C). Similarly, the overall composition of PS and LPS species did not differ significantly between the Ad‐LPLAT14 and Ad‐Luc groups (Figure 2D,E). However, the proportion of OA‐containing PS was significantly increased in the Ad‐LPLAT14 group (Figure 2F). We further evaluated the relative proportions of saturated fatty acids (SFAs), monounsaturated fatty acids (MUFAs), and polyunsaturated fatty acids (PUFAs) in PE and PS. No apparent differences were observed in the fatty acid composition of PE between the two groups (Supplemental Figure 2A). In contrast, PS in the Ad‐LPLAT14 group showed an increased proportion of MUFAs and a corresponding decrease in PUFAs compared with the Ad‐Luc group (Supplemental Figure 2B). We also analyzed phosphatidylcholine (PC) and lysophosphatidylcholine (LPC), which represent major hepatic phospholipid classes. Consistent with the findings for PE and LPE, no significant differences were observed in the overall composition of PC and LPC species between the Ad‐LPLAT14 and Ad‐Luc groups (Supplemental Figure 3A–C). These findings indicate that hepatic LPLAT14 overexpression selectively increases OA‐containing PS and shifts the fatty acid composition of PS toward a higher MUFA and lower PUFA proportion, without substantially affecting PE or PC species.

**FIGURE 2:**
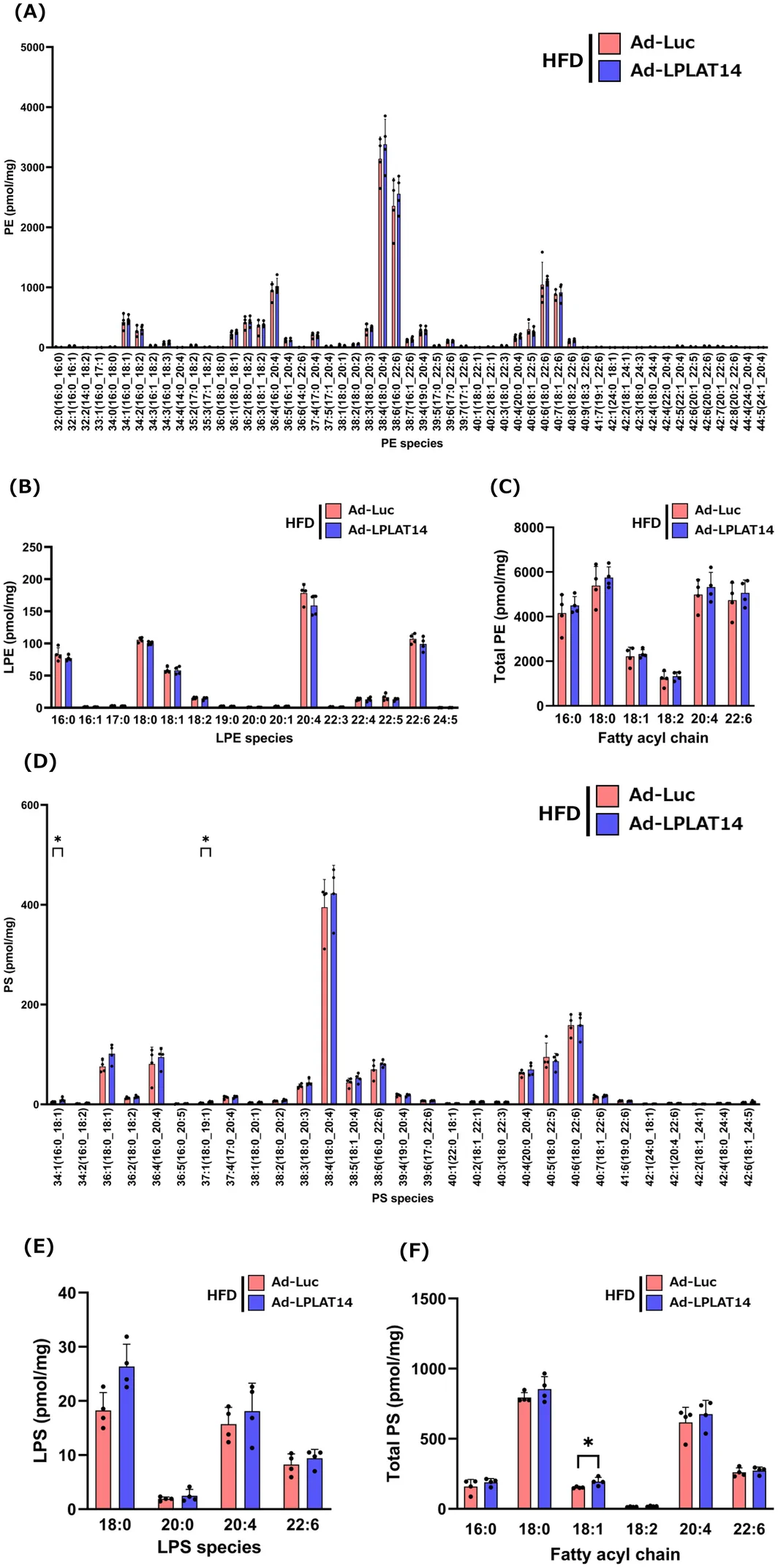
Analysis of hepatic lysophosphatidylethanolamine (LPE), phosphatidylethanolamine (PE), lysophosphatidylserine (LPS), and phosphatidylserine (PS) species. (A) PE species, (B) LPE species, (C) total PE levels containing specific fatty acyl species, (D) PS species, (E) LPS species, and (F) total PS levels containing specific fatty acyl species in mouse livers 2 weeks after administration of lysophospholipid acyltransferase‐expressing Ad vector (Ad‐LPLAT14) or a luciferase‐expressing Ad vector (Ad‐Luc), as determined by liquid chromatography–tandem mass spectrometry (LC–MS/MS). The exact sn‐positions of the acyl chains could not be definitively determined for any of the lipid species analyzed. Differences between the two independent groups were assessed using the Mann–Whitney *U* test. Data are expressed as the mean ± SE (*n* = 4 per group). *p < 0.05 versus the Ad‐Luc group.

### 3.3 The Metabolic Phenotype of Mice Treated With Hepatic LPLAT14 Overexpression Did Not Affect Glucose Metabolism

To examine the effect of LPLAT14 on body weight, the body weights of mice were measured for 8 weeks. Body weight gain was comparable among Ad‐LPLAT14‐, Ad‐Luc‐, and Phosphate‐Buffered Saline (PBS)‐treated HFD‐fed mice (Figure 3A). Next, to assess the effect of LPLAT14 on glucose metabolism, fasting blood glucose levels were measured at 2 and 8 weeks after administration of Ad‐LPLAT14, Ad‐Luc, or PBS. Fasting blood glucose levels were comparable between Ad‐LPLAT14‐ and Ad‐Luc‐treated HFD‐fed mice (Figure 3B,C). At 2 weeks after administration of Ad vectors, no significant differences in fasting insulin levels and HOMA‐IR were observed between Ad‐LPLAT14‐ and Ad‐Luc‐treated mice (Figure 3D,E). To further evaluate glucose tolerance, intraperitoneal glucose tolerance tests were performed. Blood glucose excursions did not differ significantly between Ad‐LPLAT14‐ and Ad‐Luc‐treated mice at 2 and 5 weeks after administration of Ad vectors (Figure 3F,G). Taken together, these results indicate that hepatic overexpression of LPLAT14 in mice does not affect glucose metabolism.

**FIGURE 3:**
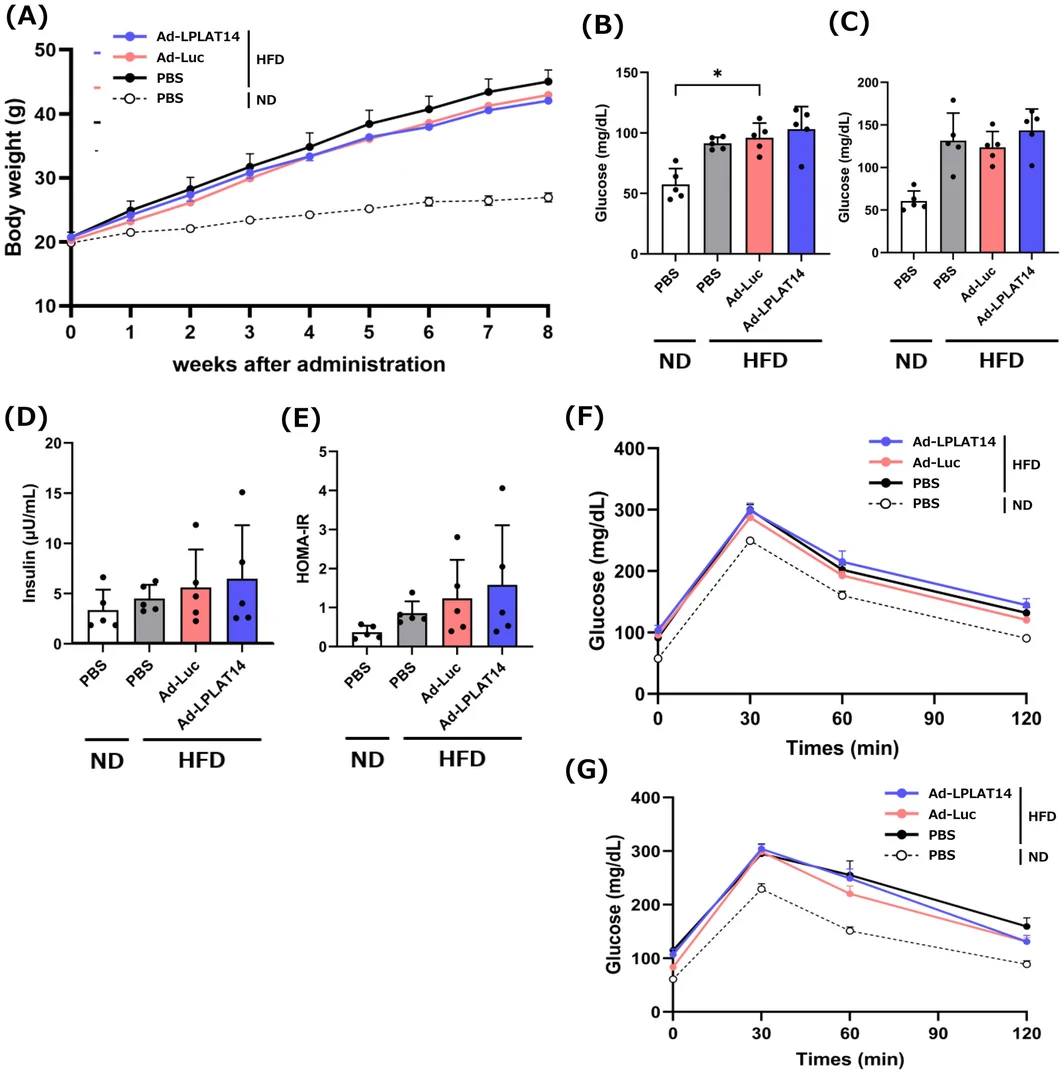
Body weight and analysis of glucose metabolism. (A) Body weights of mice fed a normal diet (ND) or high‐fat diet (HFD) and treated with an LPLAT14‐expressing Ad vector (Ad‐LPLAT14), a luciferase‐expressing Ad vector (Ad‐Luc), or phosphate‐buffered saline (PBS). Fasting glucose levels at (B) 2 and (C) 8 weeks after Ad‐LPLAT14, Ad‐Luc, or PBS treatment. (D) Fasting insulin levels and (E) homeostatic model assessment for insulin resistance (HOMA‐IR) at 2 weeks after treatment. Blood glucose levels during the intraperitoneal glucose tolerance test at (F) 2 and (G) 5 weeks after treatment. Two independent experiments were performed and yielded similar results. Multiple comparisons were performed using one‐way analysis of variance (ANOVA) followed by Dunnett's post hoc test. Data are expressed as the mean ± SE (*n* = 5 per group). *p < 0.05 versus the Ad‐Luc group.

### 3.4 Hepatic LPLAT14 Overexpression Did Not Affect Lipid Metabolism

To examine whether LPLAT14 affects lipid metabolism, histopathological analysis of liver sections was performed at 2 weeks after administration of Ad‐LPLAT14, Ad‐Luc, or PBS. Histological analysis revealed lipid droplet accumulation in the livers of HFD‐fed mice; however, no other differences were detected between Ad‐LPLAT14‐treated mice and the other HFD‐fed groups (Figure 4A). Next, to assess hepatic TG, Oil Red O staining was performed on liver sections 2 weeks after administration of Ad‐LPLAT14, Ad‐Luc, or PBS. Oil Red O staining revealed no apparent differences in lipid content between Ad‐LPLAT14‐treated mice and the other HFD‐fed groups (Figure 4B). Consistently, quantification of the Oil Red O‐positive area showed no significant differences between Ad‐LPLAT14‐ and Ad‐Luc‐treated HFD‐fed mice (Figure 4C). In addition, fasting blood TG, FFA, and CHO levels were measured at 2 weeks after the administration of Ad‐LPLAT14, Ad‐Luc, or PBS. No significant differences were observed between Ad‐LPLAT14‐ and Ad‐Luc‐treated HFD‐fed mice (Figure 4D,E). Collectively, these results indicate that hepatic overexpression of LPLAT14 in mice does not affect lipid metabolism.

**FIGURE 4:**
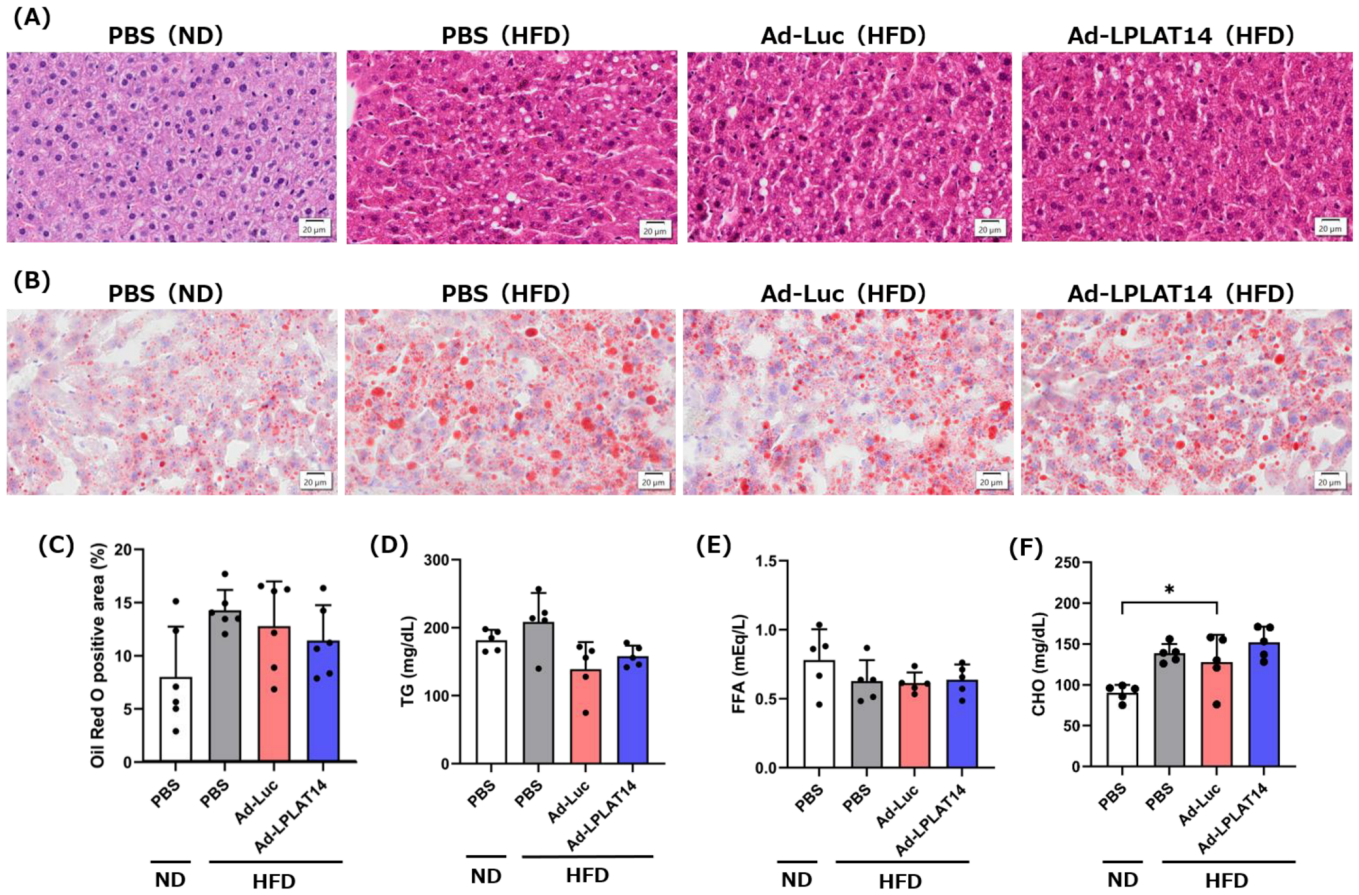
Analysis of lipid metabolism. Liver sections obtained 2 weeks after treatment with an LPLAT14‐expressing Ad vector (Ad‐LPLAT14), a luciferase‐expressing Ad vector (Ad‐Luc), or phosphate‐buffered saline (PBS) were stained with (A) hematoxylin and eosin (H&E) and (B) Oil Red O. Scale bar = 20 μm. (C) Quantification of the Oil Red O‐positive area (%). Fasting serum levels of (D) triglycerides (TG), (E) free fatty acids (FFA), and (F) total cholesterol (CHO) at 2 weeks after treatment. Two independent experiments were performed and yielded similar results. Multiple comparisons were performed using one‐way analysis of variance (ANOVA) followed by Dunnett's post hoc test. Data are expressed as the mean ± SE (*n* = 5 per group). *p < 0.05 versus the Ad‐Luc group.

### 3.5 Amelioration of HFD‐Induced Mild Liver Injury by Liver‐Specific Expression of LPAT14

In addition to metabolic profiles, we assessed the levels of HFD‐induced liver injury markers. At 2 weeks, ALT levels were significantly reduced in the Ad‐LPLAT14 group compared to the Ad‐Luc group (Figure 5A). In addition, LDH levels tended to be lower in the Ad‐LPLAT14 group than in the Ad‐Luc group (Figure 5B). At 8 weeks, both ALT and LDH levels were comparable between the Ad‐LPLAT14‐ and Ad‐Luc‐treated groups (Figure 5C,D). Collectively, these results indicate that liver‐specific overexpression of LPLAT14 ameliorates mild liver injury during early HFD feeding.

**FIGURE 5:**
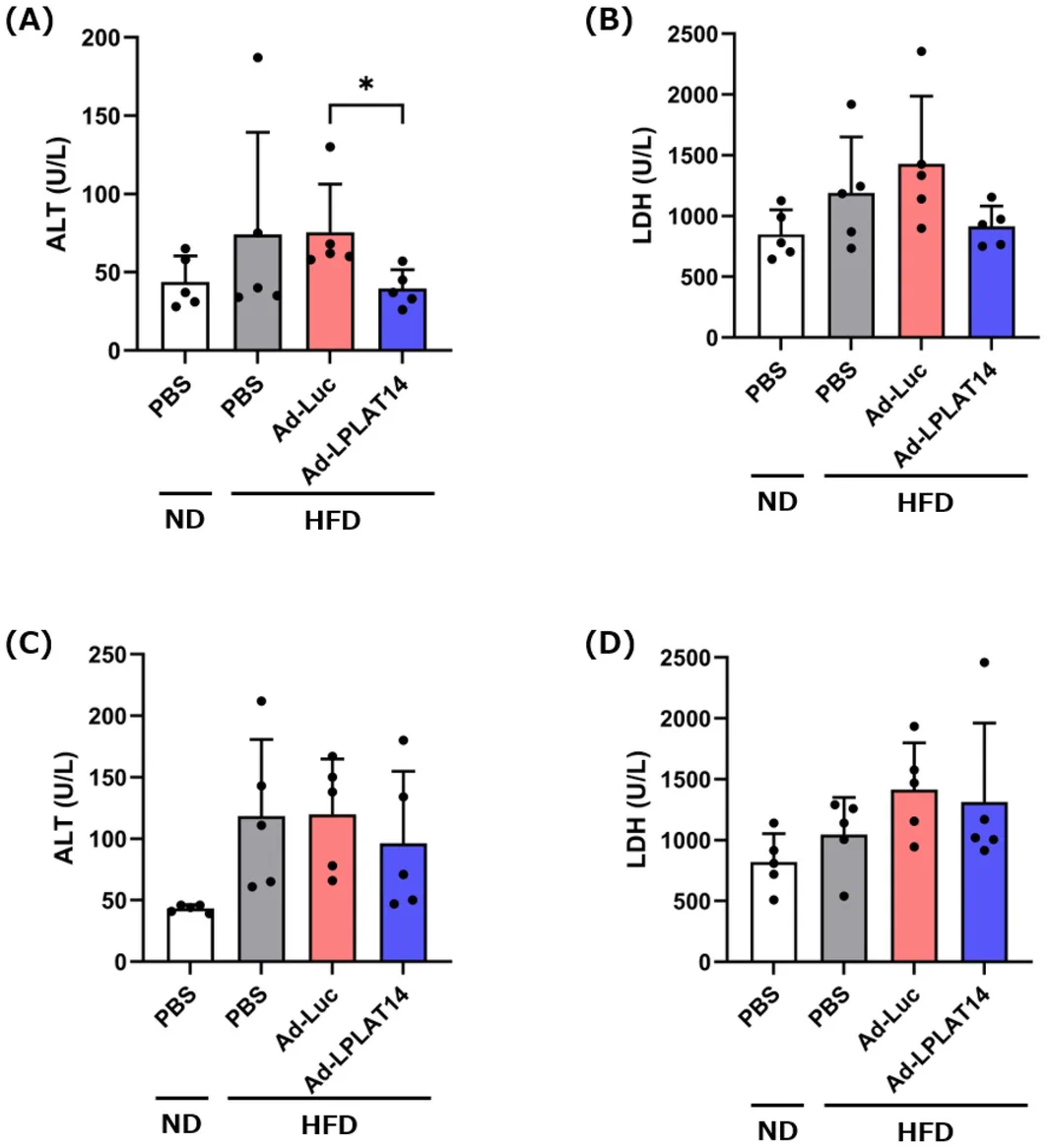
Amelioration of high‐fat diet (HFD)‐induced liver injury in mice treated with lysophospholipid acyltransferase‐expressing Ad vector (Ad‐LPLAT14). Fasting serum levels of (A) alanine aminotransferase (ALT) and (B) lactate dehydrogenase (LDH) at 2 weeks after treatment with Ad‐LPLAT14, a luciferase‐expressing Ad vector (Ad‐Luc), or phosphate‐buffered saline (PBS). Fasting serum levels of (C) ALT and (D) LDH at 8 weeks after treatment. Two independent experiments were performed and yielded similar results. Multiple comparisons were performed using one‐way analysis of variance (ANOVA) followed by Dunnett's post hoc test. Data are expressed as the mean ± SE (*n* = 5 per group). *p < 0.05 versus the Ad‐Luc group.

## 4 Discussion

We investigated the effect of hepatic overexpression of LPLAT14 on the metabolic alterations resulting from an HFD. Our results revealed that hepatic overexpression of LPLAT14 did not significantly affect glucose and lipid metabolism parameters but attenuated HFD‐induced mild liver injury. Furthermore, hepatic overexpression of LPLAT14 altered PS and LPS species and increased the levels of OA‐containing PS. Our results indicate that LPLAT14‐mediated increases in the levels of OA‐enriched PS may protect against HFD‐induced mild liver injury.

No significant differences were found in fasting blood glucose levels or glucose tolerance between Ad‐LPLAT14‐ and Ad‐Luc‐treated mice (Figure 3). In addition, indicators of lipid metabolism, including serum markers, liver histology, hepatic lipid accumulation, and lipid metabolism‐related gene expression showed no variation (Figure 4). These findings are likely attributable to the fact that the composition of major hepatic phospholipids, such as PC, LPC, PE, and LPE species was not altered (Figure 2).

While metabolic markers remained unchanged, serum ALT levels, which serve as a representative phenotype reflecting early‐stage mild liver injury in our 2‐week short‐term HFD model, were significantly reduced by hepatic overexpression of LPLAT14. We speculate that this hepatoprotective effect may be associated with inhibition of ferroptosis. LPLAT14 has been reported to protect against ferroptosis [25, 26, 27]. Mechanistically, LPLAT14 incorporates OA, a monounsaturated fatty acid, into membrane phospholipids [28, 29, 30]. This remodeling replaces polyunsaturated fatty acids, the primary substrates for lipid peroxidation, thereby limiting the propagation of oxidative damage. In this study, we found that hepatic overexpression of LPLAT14 increased the level of OA‐containing PS in the liver.

Phospholipid diversity arising from variations in polar head groups and fatty acids, as regulated by LPLATs, has been reported [18]. However, phospholipid profiles determined by LPLAT14 in vivo have not yet been elucidated. Our study provides the first characterization of PC, LPC, PE, LPE, PS, and LPS profiles associated with hepatic LPLAT14 overexpression in mice using an Ad vector‐mediated overexpression system. LPLAT14 was found to incorporate OA into PS. Our data are consistent with a previous study demonstrating that LPLAT14 exhibits endogenous activity toward incorporating OA into LPS in vitro [23, 24]. In contrast, our findings regarding OA‐containing PE differed from previous in vitro findings [24]. This discrepancy may be attributed to the differences between in vitro and in vivo experimental conditions. Additionally, because the HFD used in this study was enriched in OA and LPLATs are abundantly expressed in the liver, OA‐containing PE is likely to accumulate as a background lipid species. In this context, the LPLAT14‐mediated increase in OA‐containing PE might be less apparent due to a more dominant increase in OA‐containing PE synthesized by other LPLATs, such as LPLAT12 (LPCAT3), which are highly expressed in the liver and incorporate OA into LPE [23, 42].

Although we demonstrate that hepatic LPLAT14 overexpression attenuates mild liver injury during early HFD feeding by enriching OA‐containing PS, some limitations remain. The precise molecular mechanisms linking this lipidomic alteration to hepatoprotection, as well as the reason this effect is restricted to the early phase (2 weeks) and disappears by 8 weeks, remain unclear. We speculate that ferroptosis suppression may be involved, as MUFA accumulation is a known anti‐ferroptotic signal. This hypothesis is supported by our finding that the Ad‐LPLAT14 group exhibited an increased proportion of MUFAs and a corresponding decrease in PUFAs specifically within the PS fraction. Dietary MUFAs, predominantly oleic acid, have themselves been reported to suppress ferroptosis. We hypothesize that this protective effect may be enhanced when MUFAs are incorporated into membrane phospholipids, such as PS, and that LPLAT14 may contribute to this process. Thus, rather than masking the effect of LPLAT14, abundant dietary oleic acid may serve as a substrate for LPLAT14‐mediated remodeling of membrane phospholipids. Such remodeling may increase MUFA incorporation into phospholipids and thereby contribute to resistance to ferroptosis. Although ferroptosis has been implicated in liver injury in chronic HFD models [43, 44, 45, 46], its relevance at the early 2‐week stage remains unclear. Evaluation of ferroptosis at this stage is challenging because of the limited availability of robust in vivo assays and the relatively mild liver injury observed in this short‐term model. Furthermore, advanced histopathological changes generally require longer periods of HFD feeding or additional stressors, such as CCl_4_, which represents a limitation of our 2‐week model. Nevertheless, successful high‐fat loading within this short timeframe was supported by hepatic lipid accumulation, as demonstrated by Oil Red O staining, and elevated serum cholesterol levels. Accordingly, we consider the increase in serum ALT to represent an early‐stage phenotype of HFD‐induced mild liver injury in this model. Future studies are warranted to characterize the precise nature of liver injury at Week 2 and to clarify how the underlying pathogenesis evolves by Week 8. In addition, because our findings were obtained in HFD‐fed mouse models, they may not fully recapitulate human disease. Studies using human clinical samples will therefore be required to determine the translational relevance of these findings.

In summary, our study demonstrates that hepatic LPLAT14 overexpression in mice is associated with attenuation of mild liver injury and an increase in hepatic OA‐containing PS levels. These findings suggest that LPLAT14‐mediated phospholipid remodeling may contribute to hepatoprotection and that LPLAT14 may represent a potential therapeutic target for mild liver injury.

## Author Contributions

Sho Hori conceived and designed the project, performed the experiments, analyzed the data, and wrote the manuscript. Kahori Shimizu conceived and designed the project, performed the experiments, revised the manuscript, and supervised the project. Taiga Kikuchi and Hajime Miyata performed the experiments. Hiroaki Hase and Hiroki Nakanishi performed and analyzed the lipid measurements. Hideo Shindou revised the manuscript. Hiroyuki Mizuguchi revised the manuscript and supervised the study. All authors approved the manuscript.

## Ethics Statement

All animal procedures were approved by the Institutional Animal Care and Use Committee of Osaka University (approval ID doyaku R06‐3), and they were performed in accordance with the Institutional Guidelines and Regulations for Animal Experiments at the University of Osaka.

## Conflicts of Interest

The authors declare no conflicts of interest.

## Supporting information


**Supplemental Figure 1:** Fatty acid composition of the HFD32 diet. (A) Relative abundance (%) of individual fatty acids in the HFD32 diet. (B) Relative proportions (%) of saturated fatty acids (SFAs), monounsaturated fatty acids (MUFAs), and polyunsaturated fatty acids (PUFAs) in the HFD32 diet.

**Supplemental Figure 2:** Distribution of saturated, monounsaturated, and polyunsaturated fatty acids in hepatic phospholipids. Pie charts showing the relative proportions (%) of saturated fatty acids (SFAs), monounsaturated fatty acids (MUFAs), and polyunsaturated fatty acids (PUFAs) in hepatic phospholipids. (A) Fatty acid composition of phosphatidylethanolamine (PE) in the Ad‐Luc and Ad‐LPLAT14 groups. (B) Fatty acid composition of phosphatidylserine (PS) in the Ad‐Luc and Ad‐LPLAT14 groups. Data are presented as the mean percentage of total fatty acids (*n* = 4 per group).

**Supplemental Figure 3:** Analysis of hepatic phosphatidylcholine (PC) and lysophosphatidylcholine (LPC) species. (A) PC species, (B) total PC levels containing specific fatty acyl species, and (C) LPC species in mouse livers 2 weeks after administration of an LPLAT14 expressing adenoviral vector (Ad‐LPLAT14) or a luciferase‐expressing adenoviral vector (Ad‐Luc), as determined by liquid chromatography–tandem mass spectrometry (LC–MS/MS). The exact sn positions of the acyl chains could not be definitively determined for any of the lipid species analyzed. Differences between the two independent groups were assessed using the Mann–Whitney U test. Data are expressed as the mean ± SE (*n* = 5 per group). *p* < 0.05 versus the Ad‐Luc group.

## Data Availability

The data that support the findings of this study are available in the Materials and Methods, Results, and Supporting Information of this article.
