# Supplementary material for: Hepatic Overexpression of Lysophospholipid Acyltransferase LPLAT14/MBOAT1 Ameliorates Mild Liver Injury During Early High‐Fat Diet Feeding
Source: FASEB Bioadv. 2026 Nov 2;8(11):e70165. doi: 10.1096/fba.2026-00165 (PMC13725374; doi:10.1096/fba.2026-00165)

Supply Figure 3

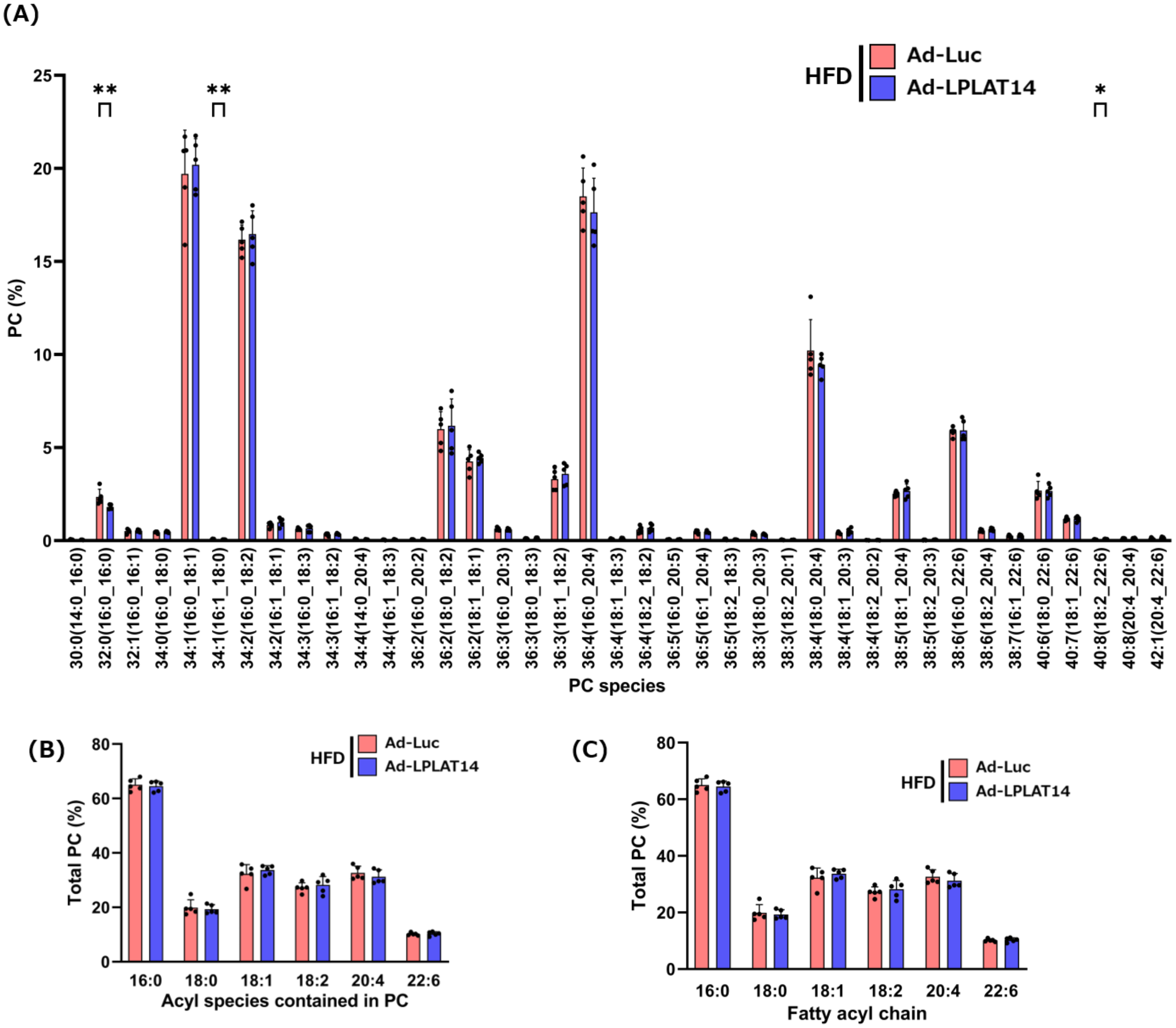

Supplement: Supplementary file 2 [file FBA2-8-e70165-s002.pdf]
