# Supplementary material for: Hepatic Overexpression of Lysophospholipid Acyltransferase LPLAT14/MBOAT1 Ameliorates Mild Liver Injury During Early High‐Fat Diet Feeding
Source: FASEB Bioadv. 2026 Nov 2;8(11):e70165. doi: 10.1096/fba.2026-00165 (PMC13725374; doi:10.1096/fba.2026-00165)
Supplement: Supplementary file 3 [file FBA2-8-e70165-s003.pdf]

Supply Figure 1

(A)

Fatty acid		%
Myristic acid	14:0	1.1
Myristoleic acid	14:1	0.3
Pentadecanoic acid	15:0	0.1
Palmitic acid	16:0	12.6
Palmitoleic acid	16:1	1.2
Heptadecanoic acid	17:0	0.4
Heptadecenoic acid	17:1	0.3
Stearic acid	18:0	7.5
Oleic acid	18:1	64.3
Linoleic acid	18:2	10.2
Linolenic acid	18:3	0.2
Arachidic acid	20:0	0.3
Eicosenoic acid	20:1	0.3
Behenic acid	22:0	0.2
Unidentified	ー	0.8
Total	ー	100.0

(B)

Classification of fatty acid	%
Saturated Fatty Acid	22.3
Monounsaturated Fatty Acid	66.5
Polyunsaturated Fatty Acid	10.4
Unidentified	0.8
Total	100.0
